# Comparative analysis of ESTs involved in grape responses to *Xylella fastidiosa *infection

**DOI:** 10.1186/1471-2229-7-8

**Published:** 2007-02-22

**Authors:** Hong Lin, Harshavardhan Doddapaneni, Yuri Takahashi, M Andrew Walker

**Affiliations:** 1 USDA-ARS, 9611 S. Riverbend Avenue, Parlier, California 93648, USA; 2 Department of Viticulture & Enology, University of California, Davis, California 95616, USA; 3 Department of Food sciences, Ehime Women's College, Uwajima, Ehime 798-0025, Japan

## Abstract

**Background:**

The gram-negative bacterium *Xylella fastidiosa *(Xf) is the causal agent of Pierce's disease (PD) in grape as well as diseases of many fruit and ornamental plants. The current molecular breeding efforts have identified genetic basis of PD resistance in grapes. However, the transcriptome level characterization of the host response to this pathogen is lacking.

**Results:**

Twelve tissue specific subtractive suppression hybridization (SSH) cDNA libraries derived from a time course sampling scheme were constructed from stems, leaves and shoots of PD resistant and susceptible sibling genotypes (*V. rupestris *× *V. arizonica*) in response to Xf infection. A total of 5,794 sequences were obtained from these cDNA libraries from which 993 contigs and 949 singletons were derived. Using Gene Ontology (GO) hierarchy, the non-redundant sequences were classified into the three principal categories: molecular function (30%), cellular components (9%) and biological processes (7%). Comparative analysis found variations in EST expression pattern between infected and non-infected PD resistant and PD susceptible grape genotypes. Among the three tissues, libraries from stem tissues showed significant differences in transcript quality suggesting their important role in grape-*Xylella *interaction.

**Conclusion:**

This study constitutes the first attempt to characterize the *Vitis *differential transcriptome associated with host-pathogen interactions from different explants and genotypes. All the generated ESTs have been submitted to GenBank and are also available through our website for further functional studies.

## Background

Pierce's disease (PD) has been a chronic problem for California's grape industry since the 1880s. The threat from this disease has recently become more severe with the introduction and establishment of a more effective vector, the glassy-winged sharpshooter (*Homalodisca coagulate*). The disease is caused by *Xylella fastidiosa*, a xylem-limited, gram negative bacterium that is hosted by a wide range of plant species in and around vineyards in the southern United States and Mexico [1]. Over the past few years, federal, state governments, and the grape industry have funded PD research. Much of this research has focused on means of controlling the vector with insecticides and natural predators as a critical first step in integrated crop management. However, even low populations of the glassy-winged sharpshooter can have severe impact on vineyard health, thus limiting the effectiveness of predators to solve PD. In addition, as pesticide use becomes more restricted and as pesticide resistance develops, it is likely that the ultimate solution to PD will be host resistance.

Resistance to PD exists in some grape species and cultivars have been bred from these species. For example, accessions of *Vitis aestivalis*, *V. arizonica, V. shuttleworthii, and V. smalliana *are highly resistant to PD [2], and breeding programs have utilized these resistant species to develop PD resistant grapes for the southeastern United States [3]. Efforts to breed PD resistant grapes for California are underway [4]. The goals of these breeding efforts are to develop durably resistant cultivars, map and identify DNA-based markers for resistance to aid in selection, and to identify resistance genes. The introduction of PD resistance genes into wine grapes is complicated by the need for several generations of back-crossing to exclude unfavorable fruit characters associated with the resistant *Vitis *species. Once resistance genes are identified it may be possible to directly introduce resistance into elite wine grape cultivars by transgenic technologies.

Systemic infection studies under greenhouse conditions have shown differential distribution patterns of *X. fastidiosa *populations between resistant and susceptible genotypes and also among different organs or tissues of resistant genotypes [2]. This study found that *X. fastidiosa *populations in the tissues of susceptible genotypes did not differ among nodes, internodes, petioles, and leaf blades. However, the resistant genotypes had lower *X. fastidiosa *population levels, with highest levels in leaf blades, followed by petioles, and lowest levels in stem nodes and internodes. Differences between *X. fastidiosa *populations in the resistant genotypes compared to the susceptible genotypes were greatest in the stem internodes. The inheritance of PD resistance in a *V. rupestris *× *V. arizonica *population was also evaluated by quantifying *X. fastidiosa *levels with ELISA [5] and by symptomology, including leaf scorch and a cane maturation index [2]. From genotypic screening and genetic mapping studies, it was concluded that a dominant allele controls PD resistance [5]. More recently, Krivanek et al. [6] have identified a locus that is linked to PD resistance and denoted it as 'Pierce's disease resistance 1' (*PdR1*). These studies confirm that there is genetically based PD resistance in grapes. They also found a range of resistance and tolerance to *X. fastidiosa*, which suggests that host responses to the pathogen are genotype dependent. The results from these studies prompted investigations into molecular basis of these host-pathogen interactions, which are currently poorly understood.

Functional genomic approaches provide powerful tools for identifying expressed genes. Among these techniques, expressed sequence tags (EST), [7], serial analysis of gene expression (SAGE), [8] and massively parallel signature sequencing (MPSS), [9], have been successfully employed. However due to its relative simplicity and ease, single pass EST sequencing has been the most widely used method to characterize genes associated with cellular development, biotic and abiotic stress in plant research.

Subtractive suppression hybridization (SSH) EST cloning can be used to maximize the identification of genes involved in host responses to pathogen infection and disease development. SSH cloning is also an effective method for cloning differentially regulated genes in cells. This technique has been used to isolate plant genes that are expressed in response to infection [10-12]. Using molecular hybridization and subtraction techniques, the SSH cDNA library approach reduces the cloning of abundantly expressed housekeeping genes or genes commonly expressed in both control and treated plants, thereby normalizing expressed cDNA profiles during library construction. As a result, it significantly enhances the chances of cloning differentially expressed genes. This is particularly important because many pathogenesis-related genes are expressed at low levels, and can be limited to a particular tissue or cell type [13]. These genes are less likely to be represented in a library if standard EST cloning methods are used. Recently completed EST projects have greatly contributed to the total number of developmentally regulated *Vitis *ESTs available in the public domain [14-16]. Further, there is information on microarray gene expression associated with viral infection [17] and on individual ESTs involved in host defense such as nonspecific lipid-transfer proteins (nsLTPs) [18] and phytoalexin [19]. However, to date information on ESTs expressed in response to the *X. fastidiosa *challenge is lacking.

The goal of this study was to characterize the molecular events in the grape/*X. fastidiosa *interaction using the SSH technique to compare populations of mRNA from highly resistant and susceptible grape genotypes from a grape mapping population being used to characterize PD resistance derived from a *V. arizonica *× *V. candicans *hybrid [5]. For instance, the identified putative genes that are associated with host defense and/or resistance responses in this study can be used to develop molecular markers for PD resistance genetic mapping project. They are also useful for molecular-assistant-selection if they are found to be tightly linked to the PD resistance genes. To maximize cloning expression profiles associated with the host-pathogen interaction, a time course sampling scheme was designed tissue specific cDNA libraries were constructed from stem, leaf and shoot tissues. This report provides the transcriptome analysis of contrasting genotypes in response to *X. fastidiosa *infection among different tissues and provides ESTs associated with this host-pathogen interaction.

## Results

### Sequencing and assembly

A total of 5,794 ESTs with an average of 482 ESTs per library were sequenced from the 12 SSH libraries. The average size of the EST was 282 bp with 5,421 sequences of 100 bp or more. The number of ESTs sequenced from each library varied from 290 to 715 sequences (Table 1). Transcript redundancy in the EST collection was reduced by first comparing clusters within each library and then among all 12 libraries. These comparisons resulted in the assembling of 1,942 unique sequences including 993 clusters (contigs) and 949 singleton ESTs (Table 1). The percentage of unique sequences in each library varied from 19.3 to 74.5% (Table 1). In the resistant genotype 9621-67, transcript diversity from leaf and shoot tissues was reduced from 74.5 to 28.96% and from 37.96 to 21.4%, respectively, after infection by *X. fastidiosa*. However the opposite results were observed in the stem tissue where transcript diversity increased from 43.3 to 57.7% (Table 1). In the susceptible genotype, on the other hand, transcript diversity was reduced in infected leaf and stem tissues and also in the non-infected shoot tissue (Table 1).

In order to assess the number of unique and overlapping transcripts among the 12 libraries, four comparisons were made: those derived from resistant infected (RI)-libraries (libraries, 1, 2 and 9); those derived from resistant control (RC)-libraries (libraries, 4, 5 and 11); those derived from susceptible infected (SI)-libraries (libraries, 3, 7 and 12); and those from susceptible control (SC)-libraries (libraries, 6, 8 and 10).

**Figure 1:**
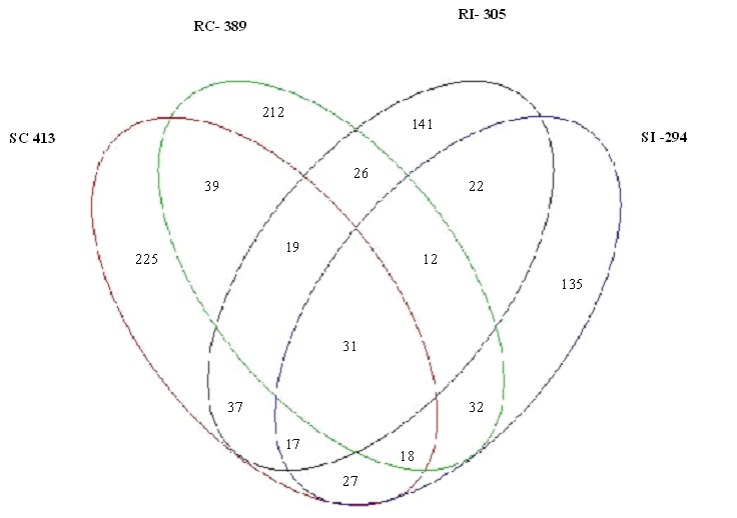
**Co-expression pattern of the ESTs**. The 12 SSH libraries were grouped into four classes: resistant infected (RI)-libraries 1, 2 and 9; resistant control (RC)-libraries 4, 5 and 11; susceptible infected (SI)- 3, 7 and 12; and susceptible control (SC)- 6, 8 and 10 libraries. The distribution of the ESTs that were used to generate the 993 non-redundant contigs was plotted among the four classes.

There were a total of 1561 contigs 338, 440, 336 and 447 that were further assembled into the four respective classes 305 (RI), 389 (RC), 294 (SI) and 413 (SC). These sequences were later used to construct the 993 non-redundant contigs for all 12 libraries (Table 1). Singletons were not included in this analysis. Contigs were grouped as present in one, two, three or all the four classes (Figure 1).

The number of non-overlapping sequences in the above four classes was 141 (RI), 212 (RC), 135 (SI) and 225 (SC), respectively. Only 31 sequences were common among all four classes; 39 contigs had ESTs that were expressed in the two control classes (RC and SC) and 22 had ESTs common between the two infected classes (RI and SI) (Figure 1). The distribution also included 32 contigs that were made from SI and RC classes and 37 contigs that were made from RI and SC classes. After this analysis, 72% of the 993 unique contigs belonged to one of the above four class, while the remaining 28% were overlaps.

### Functional annotation of the ESTs and comparative expression analysis

Comparison of the 1,942 non-redundant sequences from the SSH libraries against the non-redundant protein database (nr) of the NCBI revealed that 716 sequences have significant similarity (≤ 1E^-5^) to existing sequences and 1,226 were unique. Only two ESTs showed significant similarity to *X. fastidiosa *(Additional file 1). Complete details of the blast results are available through our website [20].

**Figure 2:**
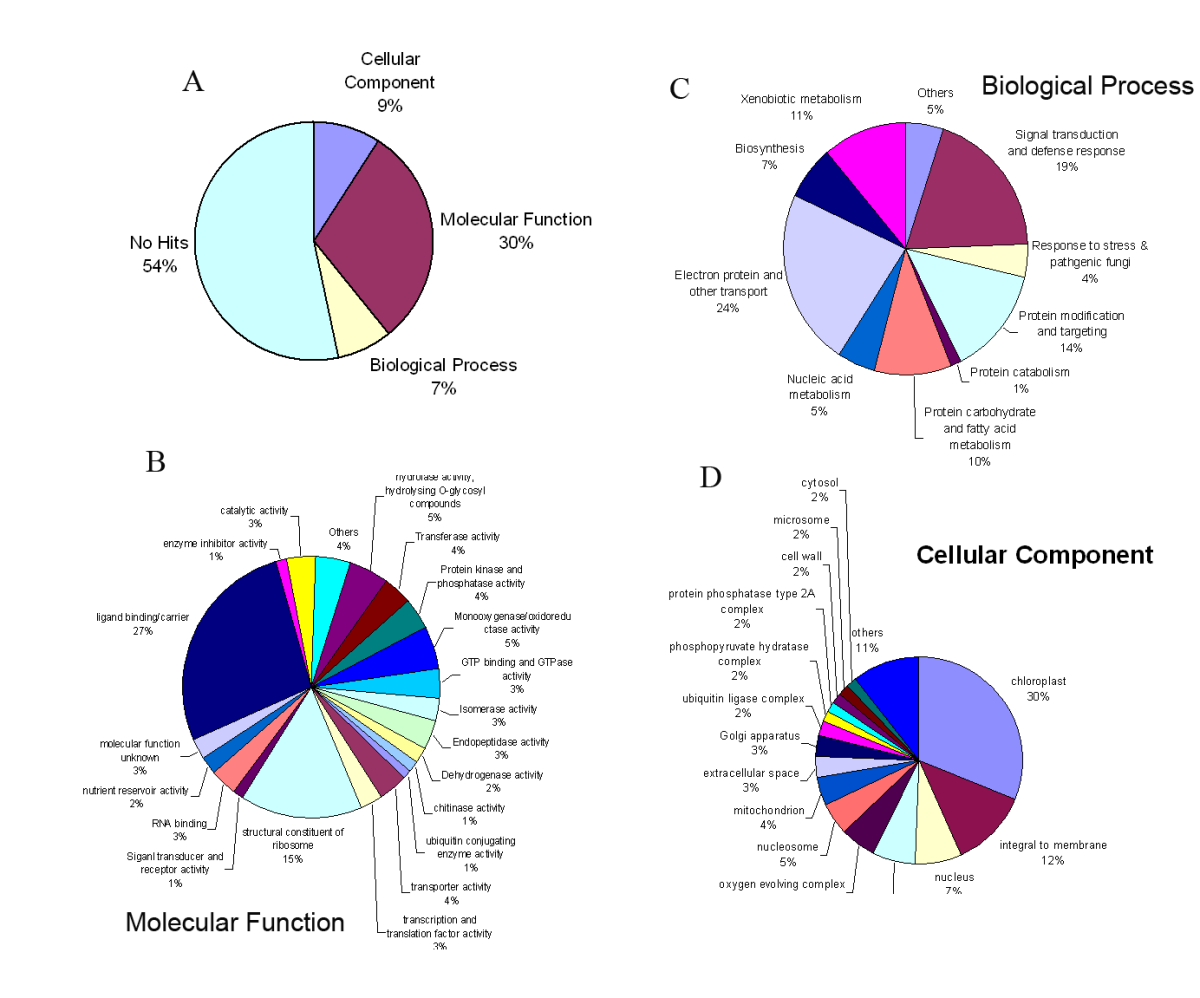
Percentage representation of gene ontology (GO) mappings for the 9621-67 and 9621-94 hybrids clusters. Functional annotation was carried using the High Throughput Gene Ontology Functional Annotation (Ht-Go-Fat) toolkit. The pie diagrams show the distribution of 905 sequences among the three principal GO categories. EST distribution (A) among the three GO principles (B) Molecular Function (C) Biological Process (D) Cellular Component.

When these 1,942 sequences were passed through the Ht-Go-Fat toolkit and BLAST searched against the supplied database, 915 sequences generated a hit, out of which 904 had at least one GO term (Additional file 2). Based on the generated GO information, these 904 sequences were divided in to the three principal GO categories: molecular function (30%), cellular component (9%) and biological process (7%) (Figure 2A). Under the molecular function category, ligand binding and carrier protein contributed for 27% of the total contigs followed by the ribosomal coding transcripts 15% (Figure 2B). Transport sequences 24% followed by signal transduction and defense response sequences 19% accounted for the majority of those in the biological process category, while many of the sequences in the cellular component category were in the chloroplast 30%, membrane and nucleus subsections 26% (Figure 2C&2D). More than half of the sequences (54%) did not match sequences in the existing databases (Fig 2A) and other sequences were divided among the three principle categories. The full list of gene annotation along with the corresponding GO terms can be queried through our website [20].

Non-redundant sequences (contigs and singleton ESTs) from each individual library were analyzed using the GO classification. In order to address the issue of uneven EST numbers from each library, we compared relative abundance of the gene function categories based on their relative proportions from different library types (Table 2). Among the leaf tissue libraries, the non-infected leaf RC library was significantly different from the other leaf libraries because of the higher percentage of ESTs representing signal transduction and defense response (6.5), xenobiotic metabolism (3.25), nutrient reservoir activity (3.25), hydrolase activity and hydrolyzing O-glycosyl compounds (2.44). Infected leaf (RI) libraries showed multi-fold over expression of the monoxygenase/oxydoreductase activity (10.17 – 10.58) related ESTs compared to the non-infected leaf libraries (Table 2).

Comparison of the four stem libraries showed that the SI library differed significantly for ESTs related to xenobiotic metabolism (13.27), nutrient reservoir activity (7.08) and monooxygenase/oxidoreductase activity (3.54). In contrast, ligand binding/carrier EST category was markedly lower than for that of the other three libraries. Control libraries from both the genotypes had a higher percentage of the transport related ESTs (3.13–3.54) compared to the infected libraries (Table 2).

Among the four shoot libraries, the RI was significantly different for ESTs of protein modification and targeting (2.56) and monooxygenase/oxidoreductase activity (6.84) in comparison to the other three libraries while the SI shoot library differed for protein kinase and phosphatase activity ESTs compared to the other three libraries.

Interestingly, stem libraries showed a higher percentage of the signal transduction and defense-related response ESTs than the leaf and shoot libraries (Table 2). With the exception of the RC leaf library, chloroplast related ESTs were abundant in the leaf libraries.

In order to evaluate the diversity and specificity of the transcripts that were specific to a physiological condition, individual library specific ESTs were studied. There were 949 singleton ESTs and 689 contigs that fell into this category. The stem libraries showed reduced transcript diversity following *X. fastidiosa *infection in both the resistant (Lib-5 and Lib-2) and susceptible selections (Lib-3 and Lib-6). While the control libraries had a wide range of functional ESTs including pathogen related (PR) proteins in both the selections, infected libraries were enriched with PR proteins. The resistant infected libraries also were more diverse than the susceptible infected libraries. The resistant stem infected library had transcripts encoding PR protein such as β 1–3 glucanase and 14 kDa proline-rich protein, primary cell wall modifying proteins such as, xyloglucan endotransglycosylase (XET), endoxyloglucan transferase (EXT), and metabolic enzymes such as cinnamoyl-CoA reductase, isopropylmalate dehydrogenase, glutamate decarboxylase, 3-hydroxybutyryl-CoA dehydrogenase, PEP carboxylase, quinine reductase, and auxin responsive factor that appeared following *X. fastidiosa *infection. On the other hand, the susceptible infected stem library was over represented by transcripts encoding PR proteins such as PR-23S NP24 protein precursor and osmotin-like protein TPM-1, glucan 1,3-beta-glucosidase, seed storage legumin like protein and proteolytic pathway proteins such as aspartic protease, beta7 proteasome subunit and 20S proteasome beta subunit (PBG1) that were absent in the control library. The infected leaf libraries were free of any known transcripts of PR proteins, with control libraries having a greater percentage of transcripts encoding unknown proteins compared to the infected libraries. Only the SI shoot library had pathogen responsive ESTs (a chitinase-like protein, a nonspecific lipid-transfer protein precursor (LTP) and an F-box/LRR-repeat protein-20). The RI shoot library did not have any of the above transcripts in this given transcriptome set.

### Real-Time Quantitative RT-PCR analysis of the differential expression

RT-PCR analysis of 7 out of the 8 selected ESTs confirmed differential expression under the conditions studied. Four out of the eight ESTs had greater expression in the susceptible variety, with gradual accumulation of the transcript as the disease progressed (Table 3). Expression of these ESTs was much higher in the stem tissue than in the leaf tissue, particularly at 8-weeks post inoculation. Two of these ESTs were annotated as encoding PR proteins, while the other two appeared to be novel (Table 3). Three transcripts involved in the cell homeostasis, two belonging to the metallothionin family and a SOS2 protein kinase that is required for sodium and potassium ion homeostasis and salt tolerance in plants, showed a different trend. Expression of both the ESTs of metallothionin family was down regulated in the stem tissue at 8 weeks after inoculation in both the susceptible and resistant genotypes, while the response varied for other stages suggesting different functional roles for these two transcripts. In contrast, the expression of SOS2 protein kinase EST did not vary significantly in this process (showed less that 2-fold variation). Expression of the L11_67_Sh_CT was down regulated (-4.48 ± 2.02) in the resistant 9621-67 stem samples collected 24h post inoculation. This EST had sequence similarity to mitogen-activated protein kinase kinase (MAPKK) that was cloned from the control RC shoot library of the same genotype.

## Discussions and Conclusion

This study constitutes the first genome-wide effort to understand the molecular basis of a host-*X. fastidiosa *interaction in *Vitis*. Twelve forward and reverse suppression subtractive cDNA libraries from two genotypes (resistant and susceptible) for three different tissues and 10 different stages of Pierce's disease development were constructed to identify spatial and temporal transcriptional changes resulting from *X. fastidiosa *infection. Because a whole *Vitis *genome sequence is not yet been completed, ESTs could serve as an efficient alternative approach to the discovery of novel genomic information. Out of the 1,942 non-redundant ESTs that were cloned in this study, about 33% were found to be unique, demonstrating the effectiveness of the experimental design and the construction strategy utilized for these SSH libraries. RT-PCR analysis of seven out of the eight selected ESTs from SSH confirmed their differential expression under the test conditions. Five out of the six transcripts showed up regulation in the tissue types and condition from which they were cloned. However, the number of transcripts that were cloned for each of these ESTs (based on the ESTs that were used in generating the contig) was several folds lower than their original numbers (as indicated by the RT-PCR change values) in the RNA pool, indicating that suppression of the EST numbers that appeared in the final pool was effective. Furthermore, more than half (54%) of these sequences did not match the sequences in the GenBank and 508 were not reported in the *Vitis *EST database collection and are therefore unique contributions to the *Vitis *EST pool. A significant difference in the number and diversity of transcripts was observed in response to *X. fastidiosa *infection in the resistant vs. susceptible genotypes, suggesting host responses to infection are genotype dependent. The present study identified a group of transcripts that are regulated in response to *X. fastidiosa *infection and may represent the key elements in development of the defense response.

There was a significant reduction in transcript diversity, particularly in leaf tissues, in both the resistant and susceptible genotypes, after infection with *X. fastidiosa *(Table 1). This transcript variation was supported by the co-expression pattern of the ESTs with only 28% of the ESTs overlapping among the four classes and the rest being unique to each of those classes (Figure 1). The large percentage of transcripts involved in ligand binding, carrier signal transduction, and defense response among the annotated transcripts from the inoculated tissue also supports the presumption that many of these transcripts are specifically involved in the *X. fastidiosa *resistance response. These observations are consistent with previously reported studies on host-pathogen interactions [21,22].

Among the three tissue types, comparisons between libraries from resistant and susceptible infected stem tissues produced the most interesting EST expression patterns. The resistant library had ESTs with primary cell wall modifying and metabolic enzymes and for known PR proteins such as β 1–3 glucanase.

Plant cell elongation depends on physical properties of the primary cell wall. The class of enzymes, called alternatively endo-xyloglucan transglycosylase (EXT) or xyloglucan endotransglycosylase (XET), modifies xyloglucan (XG) by cleavage and rejoining of the β(1–4)-XG backbone. Such activity can potentially alter cell size by loosening or tightening of the cell wall. Enzymes with XET activity have been identified in rapidly growing tissues from various plant species [23] and multigene families related to XET have been identified [24,25]. Expression of primary cell wall modifying ESTs in the RI stem library, suggest active modification and expansion of cell wall tissues. Such cell wall modifications have been hypothesized to be physical barriers to limit further pathogen invasion [26]. Furthermore, expression of ESTs involved in cell metabolic activities might also reflect the pathogen's minor effect on tissue metabolism in these cells. The microarray comparative analysis study conducted by Bray [27] indicated that the xyloglucan endotransglucosylase/hydrolases (XTHs) family of genes was down regulated under water deficit conditions in three independent experiments, supporting the non-water stressed nature of the RI plants.

Enhanced transcription of β 1–3 glucanase activity in grape has been previously associated with exogenous application of ethephon, an ethylene precursor [28]. In a more recent study, Kortekamp [29] found that PR-2 (β 1–3 glucanase) expression was associated with responses to *Pseudoperenospora cubensis *infection in the resistant grape rootstock 'Gloire de Montpellier' (*V. riparia*) compared to the susceptible cultivar 'Riesling' (*V. vinifera*). EST expression in the susceptible stem library involved expression of a different class of PR proteins (PR-23S NP24 protein precursor and osmotin-like protein TPM-1) and also had different levels of seed storage and proteolytic EST expression, compared to their control tissues. Seed storage proteins such as legumins and vicillins are synthesized and accumulated during seed maturation and due to their regulation by agents such as abscisic acid, are associated with developing desiccation tolerance that occurs during seed maturation [30]. Small protein ubiquitin (Ub) and the 26S proteosome, a 2-MDa protease complex, are key components of the proteolytic pathway [31]. In response to pathogen attack, the Ub/26S proteosome pathway initiates programmed cell death to localize pathogen spread [31]. Activation of proteolysis pathway ESTs in response to the pathogen attack has been documented previously [32].

Some of the PR proteins such as chitinases and 14 kDa proline-rich protein ESTs were cloned only from resistant stem libraries. While ESTs, such as PR-10, were cloned from infected and control stem libraries of both susceptible and resistant selections. Previous reports in grape on PR-10 (intracellular proteins with unknown enzymatic function) expression point to its constitutive pre-infection role in pathogen defense [29]. The previously described proline rich proteins or P-rich proteins in *Arabidopsis *[33] and in *Drosophila *[34] are known antimicrobial compounds. Further functional studies will be required to understand the specific role of these cloned PR proteins in resistant stem tissues during *X. fastidiosa *infection.

Krivanek and Walker [2] found that resistant stems host 60-fold fewer *X. fastidiosa *cells than susceptible stems. The EST profiles produced here found unhindered metabolic activity in the resistant stem tissues and the occurrence of seed storage and proteolytic pathway proteins in the susceptible stem tissues, both suggesting the existence of a response to infection. Although PR protein expression was observed in the susceptible tissues, the nature of this expression was different since few of the PR proteins expressed in the susceptible tissues overlapped with those from resistant tissues. This finding suggests that even susceptible genotypes have a systemic and broad host defense response mechanism that responds to *X. fastidiosa *infection, it does not prevent PD and must be augmented to achieve the resistance observed in 9621-67.

Four-way comparative analysis of the *V. arizonica *hybrid sequences with three other *Vitis *species contained in the GenBank EST collections (*V. vinifera, V. shuttleworthii *and *V. aestivalis*) revealed that 26% (508 ESTs) of the *V. arizonica *sequences were unique. There are 415 ESTs in common with *V. vinifera *(Unigene Built dated 04/13/06), 57 ESTs that were present in this set and the *V. shuttleworthii *set; and 24 ESTs that were also present in *V. aestivalis *set, but absent in the other two sets. In addition, there were 338 ESTs in common with the *V. vinifera *and *V. shuttleworthii *sets; 99 ESTs that were also present in *V. vinifera *and *V. aestivalis *sets, and 14 that were present also in *V. shuttleworthii *and *V. aestivalis *sets. The rest of the ESTs were found in all four sets.

This is the first study to display the extent of EST transcript diversity in grape after infection by *X. fastidiosa*. A four-way comparative analysis found that each of the EST collections had an independent niche with varying degrees of overlap with the set produced from *V. arizonica*. This study has identified likely molecular targets for developing PD resistant varieties and for characterizing their resistance genes. Based on the diversity and specificity of the presented EST cloning results, it is clear that stem tissue plays a prominent role in the *X. fastidiosa *grape interaction, supporting observations by Krivanek and Walker [2]. The generated ESTs with its unique collection will serve as an important addition to the grape transcript pool for further large scale expression studies.

## Methods

### Plant materials and Xf inoculation experiment

Highly susceptible (9621-94) and resistant (9621-67) grape genotypes were selected from a mapping population segregating for resistance to *X. fastidiosa*. Resistance in this population derives from the *V. rupestris *× *V. arizonica/V. candicans *parent, F8909-17. This resistant selection [5] is a key parent in a PD resistance wine grape breeding program [4]. Herbaceous cuttings of both genotypes were rooted under mist-propagation and rooted plants were transplanted to 1 liter pots with a Yolo sandy loam/perlite/peat (1:1:1) soil mix. Plants were maintained in a greenhouse at 24 to 32°C with 18 hours of exposure supplemented with High-Pressure Sodium lamp (20 watts per sq. ft.). Plants were watered twice daily with 160 ml of water containing 25% Hoagland's solution (Sigma-Aldrich, St. Louis) using an automatic drip irrigation system. When plant shoots reached 30 to 40 cm, they were pruned to two basal buds before regrowth to facilitate uniform plant growth.

A *X. fastidiosa *strain obtained from the Stag's Leap district of Napa Valley, California was used to inoculate the plants. The inoculation was carried out as described previously [2] with inoculum collected from five-day-old cultures growing on PW media [35] by washing with ddH_2_O. Concentration of bacterial cells was adjusted to 6 × 10^8 ^cfu/ml (A_600 nm _= 0.25). Sixty plants from each treatment group were needle-inoculated with 10 μl of bacterial suspensions in the stem at 10 cm above the base of plants. Sixty additional plants from each treatment group were inoculated with ddH_2_O from washed sterile PW plates.

### RNA isolation and SSH cDNA library construction

Leaf, stem and shoot tip tissues were collected from five to six experimental plants at day 1, 2, and 5 post inoculation, and then at three 1-week and four 2-week intervals. All the samples were immediately stored at -80°C for later RNA extraction. PD symptoms began to develop on the susceptible genotype at about 6 weeks post inoculation and by 8 weeks, symptoms were severe. Total RNA was isolated from leaf, stem and shoot tissues using a modified CTAB extraction and lithium chloride precipitation as reported earlier [36,37]. The mRNA was isolated from total RNA using Dynabeads Oligo (dT)_25 _according to manufacturer's protocol (Dynal Biotech LLC., Brown Deer, WI USA). This step eliminated the possibility of DNA contamination in the RNA samples used for library construction. Purified mRNA samples were checked by gel and further evaluated with a BioAnalyzer. Only high quality mRNA was selected for cDNA synthesis. For each of the tissue, treatment and genotype sample, equal amounts of mRNA from time course experiments were pooled at this stage. Using 0.5 microgram of poly (A)^+ ^from each sample and SMART™ cDNA synthesis kit (BD Bioscience, Palo Alto, CA), first and second strand cDNA followed by construction of forward and reverse subtractive suppression cDNA libraries was carried out per PCR-Select cDNA Subtraction Kit's (BD Bioscience, Palo Alto, CA) protocol. At the same time, suppression subtractive hybridization was optimized and used for each library construction.

### cDNA library sequencing, data analysis and dbEST submission

**Figure 3:**
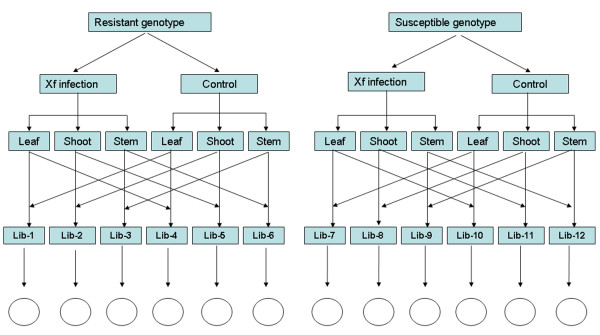
**Strategy for SSH library construction**. Twelve reciprocal tissue specific (stem, leaf and shoot) SSH cDNA libraries from highly resistant (9621-67) and highly susceptible (9621-94) sibling selections from a *Vitis rupestris *× *V. arizonica *population segregating for resistance to *X. fastidiosa *infection.

To enhance the cloning of differentially expressed genes, 12 forward and reverse SSH cDNA libraries were constructed (Figure 3). Clones were randomly selected and single-pass sequenced with a vector primer upstream of the 5'-end of the inserts. On an average, 500 clones were sequenced per library using ABI 3730 DNA Analyzer (Applied Biosystems, Foster City, CA, USA). Sequence chromatogram trace files were scored for quality with cutoff scores of Phred 20 using Base-Calling software. The FASTA files were trimmed of vector and adapter sequences. Non-target sequences such as rRNA and *E. coli *DNA were filtered out. The SSH cDNA libraries usually contained insertion sizes in a range of 200 – 700 bp. Quality sequences greater than 100 bp were selected for further analysis. Sequence files were further analyzed to determine the number of contigs and EST singletons. After contig assembly, the dataset was searched using BLASTX program against the NCBI non-redundant protein database to search for coding protein homology. This analysis resulted in 5,421 sequences that were at least 100 bp or more and had a Phred score of 20 or greater. These sequences were submitted to the GenBank EST database with the accession numbers [GenBank:DN942225 to DN947645].

ESTs were clustered and aligned into contigs and singlets using CAP3 initially from each library followed by comparison of the consensus contig sequences across all 12 libraries using MEGABLAST and grouped on the basis of similarity to clusters of related contigs. For further analysis, each cluster was represented by retaining all the consensus sequences of its contig members. Sequencing and part of contig generation were done at Macrogen Inc.

### EST Similarity search and functional assignments

A similarity search against the NCBI 'nr' database was performed for each EST sequence using the BLASTX program with a cutoff E value of 10^-4^. Functional annotation was carried using the High Throughput Gene Ontology Functional Annotation (Ht-Go-Fat) toolkit [38]. This is an ontology based database built using functional classification schemes such as Gene Ontology (GO), Enzyme Commission numbers (EC), BioCarta Pathways, and Kyoto Encyclopedia of Genes and Genomes (KEGG) pathways was downloaded from the USDA-ARS website [38] and the sequence similarity search was carried out using the default blast parameters and a cut off E value of 10^-4^.

The Gene Ontology (GO) IDs for the sequences showing a hit were then separated from the blast file and the corresponding names and ontologies were extracted from the 'Gene Ontology.obo text file' downloaded from the GO web site [39]. Next, the GO categorization scheme of classification by biological process, cellular component and the molecular function was used to categorize the similarity results and to generate the representative pie diagrams. The full set of analysis files is made available through our VitisExpDB database [20]. VitisExpDB is an online MySQL-php driven relational database that houses annotated EST data for both *V. vinifera *and non-*vinifera Vitis *species. Using the latest Gene Ontology (GO) terminology, a uniform structural vocabulary was developed to cross reference several *Vitis *accessions. The database can be searched by Gene Ontology ID, GenBank ID, enzyme number, or by inputting keyword(s).

### Real-Time quantitative RT-PCR of the differentially expressed transcripts

Gene transcripts for six of the randomly selected ESTs were quantified by real-time quantitative RT-PCR using the AB 7500 (Applied Biosystems, Foster City, CA, USA) and TaqMan One-Step RT-PCR Kit (Applied Biosystems, Foster City, CA, USA) per manufacturer's specifications. Total RNA (100 ng) extracted from leaf and stem tissues at three different stages of disease development (1 day, 3 weeks and 8 weeks post inoculation) from the susceptible 9621-94 and resistant 9621-67 was used for RT-PCR amplification using gene-specific primers (Table 4). Total RNA was normalized based on the ultra-sensitive RiboGreen^® ^RNA Quantitation Reagent (Molecular Probes, Inc. Eugene, USA) per the manufacture's protocol. This procedure was favored over gene expression based calibration because a spectrum of diseased stages vs healthy plant at different developmental stages was being compared. Furthermore, only values that were greater than 2-fold were considered significant. In the amplification reaction, SYBR Green 1 dye at 1× final concentration (10^-6 ^M) was used for quantification. Reverse Transcription was carried out at 50°C for 30 min. followed by PCR amplification for 35 cycles at 95°C for 15 sec and annealing and extension at 60°C for 1 min.

## Authors' contributions

HL conceived of the study and along with YT carried out the greenhouse experiments, RNA preparation and construction of SSH libraries. HD did the data analysis, and RT-PCR experiments. AW and HL coordinated the project and along with HD wrote the manuscript. All the authors read and approved the final manuscript.

## Supplementary Material

Additional file 1 Summary of the BLAST search results for the non-redundant set of ESTs with NCBI 'nr' database. Summary table of the NCBI BLAST search. Click here for file

Additional file 2 Summary of the GO BLAST search for the non-redundant EST set using HT-GO-FAT kit. Table with details of the GO annotation for the 915 ESTs. Sheet 2 lists the annotation details for all the 1940 sequences. Click here for file

## Figures and Tables

**Table 1:** Summary of the ESTs generated from the 12 grape SSH libraries.

Group Category	Lib I.D.	Library description	Total ESTs sequenced	Contigs	Singletons	Non-redundant ESTs	Redundant (%)	Unique (%)
**RI**	1	**infected leaf R**	487	89	15	104	78.64	21.36
	2	**infected stem R**	504	177	114	291	42.26	57.74
	9	**Infected shoot R**	404	72	45	117	71.04	28.96
**RC**	4	**non-infected leaf R**	324	95	28	123	62.04	37.96
	5	**non-infected stem R**	586	175	79	254	56.66	43.34
	11	**non-infected shoot R**	415	170	139	309	25.54	74.46
**SI**	7	**infected leaf S**	611	86	32	118	80.69	19.31
	3	**Infected stem S**	290	90	23	113	61.03	38.97
	12	**Infected shoot S**	446	160	136	296	33.63	66.37
**SC**	8	**non-infected leaf S**	589	155	160	315	46.52	53.48
	6	**non-infected stem S**	715	233	150	383	46.43	53.57
	10	**non-infected shoot S**	423	59	28	87	79.43	20.57
	**Total**		5794	1561	949			
	**Unique**			993				

The percent unique and redundant ESTs was calculated for each library. Resistant and susceptible genotypes are tagged with "R" and "S" for library description.

**Table 2:** Distribution of differentially expressed ESTs among the three tissue types. Non-redundant sequences (contigs and singleton ESTs) from each individual library were analyzed using the GO classification.

**Biological process**	**leaf**				**stem**				**shoot**			
			
	**lib-1**	**lib-4**	**lib-7**	**lib-8**	**lib-2**	**lib-5**	**lib-3**	**lib-6**	**lib-9**	**lib-11**	**lib-12**	**lib-10**
	Inf-Res	Cont-Res	Inf-Sus	Cont-Sus	Inf-Res	Cont-Res	Inf-Sus	Cont-Sus	Inf-Res	Cont-Res	Inf-Sus	Cont-Sus
Signal transduction and defense response		**6.50**		0.32	**3.09**	**3.15**	**5.31**	**2.35**			0.68	
Response to stress & pathgenic fungi			0.85		0.34	0.39		0.52		0.97	0.34	
Protein modification and targeting	1.92		**2.54**		1.37	1.97		0.78	**2.56**	0.97	0.68	
Protein catabolism										0.32	0.34	
Protein carbohydrate and fatty acid metabolism	0.96	0.81		0.63	1.03	0.39		1.04		0.65	0.68	
Nucleic acid metabolism		0.81			0.34	0.79		0.52		0.32	0.68	
Electron protein and other transport	0.96	0.81	0.85	1.27	0.69	**3.54**	0.88	**3.13**		1.62	1.69	**2.30**
Biosynthesis			1.69	0.32		0.39		0.52		0.32	1.02	
Xenobiotic metabolism		**3.25**			0.34	0.39	**13.27**	0.26		0.32		1.15
Others				0.32	0.34	1.57				0.32	0.68	
												
**Cellular component**	**leaf**				**stem**				**shoot**			
			
	**lib-1**	**lib-4**	**lib-7**	**lib-8**	**lib-2**	**lib-5**	**lib-3**	**lib-6**	**lib-9**	**lib-11**	**lib-12**	**lib-10**
Chloroplast	**11.54**		**15.25**	**11.75**	0.34		0.88	0.78	**3.42**	**2.59**	4.41	**8.05**
Integral to membrane		0.81			1.37	**4.72**	0.88	1.04		0.65	0.68	
Nucleus	**2.88**		0.85		1.37	0.39		0.78	0.85	0.32	0.34	1.15
Membrane	1.92				0.69	1.57		0.52			1.69	
Oxygen evolving complex	**2.88**		1.69	1.59				0.26		0.32	0.34	1.15
Nucleosome	**2.88**		1.69	0.32	0.69			0.52				
Mitochondrion	0.96				0.69	0.39		0.52		0.32	1.02	
Extracellular space			0.85	0.32	1.03	0.39		0.78		0.32	0.34	1.15
Golgi apparatus					0.34	0.39		0.26		0.65	0.68	
Ubiquitin ligase complex				0.63		0.39		0.26				
Phosphopyruvate hydratase complex						0.39		0.26			0.34	
Protein phosphatase type 2A complex						0.39		0.00		0.65		
												
	**leaf**				**stem**				**shoot**			
			
	**lib-1**	**lib-4**	**lib-7**	**lib-8**	**lib-2**	**lib-5**	**lib-3**	**lib-6**	**lib-9**	**lib-11**	**lib-12**	**lib-10**
Cell wall								0.52	1.71		0.34	1.15
Microsome								0.52			0.34	
Cytosol					0.34			0.26				1.15
Chromatin			0.85								0.34	
Extracellular region		1.63				0.39						
Mitochondrial inner membrane	0.96										0.34	
Plasma membrane				0.32					0.85			
Photosystem I				0.63								
Photosystem I reaction center			0.85	0.63								
Ribulose bisphosphate carboxylase complex		0.81				0.39		0.26				
Golgi membrane											0.34	
Cytoplasm								0.26				
Mitochondrial outer membrane											0.34	
Endoplasmic reticulum								0.26				
Chloroplast inner membrane											0.34	
Others												
												
**Molecular function**	**leaf**				**stem**				**shoot**			
			
	**lib-1**	**lib-4**	**lib-7**	**lib-8**	**lib-2**	**lib-5**	**lib-3**	**lib-6**	**lib-9**	**lib-11**	**lib-12**	**lib-10**
Ubiquitin conjugating enzyme activity					1.03							
Transporter activity		0.81	0.85	0.32	1.37	1.57		1.31		1.29	3.39	
Transcription and translation factor activity				0.32	1.03	0.79	2.65	0.78	1.71	1.94	1.02	1.15
Structural constituent of ribosome	4.81		**7.63**	2.54	3.44	1.57	4.42	4.70	**10.26**	**8.74**	**8.47**	**3.45**
Siganl transducer and receptor activity				0.32		0.79		0.52		0.32	1.02	
RNA binding	0.96		0.85	0.32	1.03		0.88	1.04	1.71	1.62	1.69	4.60
Nutrient reservoir activity		**3.25**					**7.08**	0.52				
Molecular function unknown	1.92	0.81	1.69		1.03	0.79	0.88	0.52		0.65	1.36	2.30
Ligand binding/carrier	**12.50**	11.38	**6.78**	3.49	**11.00**	**11.42**	3.54	**13.58**	**9.40**	6.15	**10.5**	**8.05**
Enzyme inhibitor activity		0.81		0.32	0.34	0.39		1.04		0.65		
												
	**leaf**				**stem**				**shoot**			
			
	**lib-1**	**lib-4**	**lib-7**	**lib-8**	**lib-1**	**lib-4**	**lib-7**	**lib-8**	**lib-1**	**lib-4**	**lib-7**	**lib-8**
Catalytic activity			0.85	0.32	0.34	0.79		1.04	0.85	1.62	1.69	1.15
Others	0.96		0.85	0.63	0.69	1.97	2.65	0.78	**2.56**	1.62	1.36	
Hydrolase activity, hydrolysing O-glycosyl compounds		**2.44**	0.85	0.32	2.06	1.57	0.88	1.04	**2.56**	2.27	2.03	1.15
Transferase activity		1.63	0.85	1.59	0.34	1.18	0.88	0.52	1.71	0.65	1.36	
Protein kinase and phosphatase activity	2.78		0.85		1.03	1.18		0.52	1.71	0.97	**3.39**	1.15
Monooxygenase/oxidoreductase activity	**10.58**	1.63	**10.17**	**3.17**	0.69	1.18	**3.54**	2.87	**6.84**	2.59	**2.71**	2.30
GTP binding and GTPase activity	0.96	0.81	1.69	0.32	**2.06**	1.57		1.57	1.71	1.62	**2.71**	2.30
Isomerase activity	0.96	1.63	1.69	0.32	0.69	1.57	0.88	1.57	2.56	1.62	1.02	1.15
Endopeptidase activity	**2.88**		1.69		2.06		**3.54**	0.78			1.02	**3.45**
Dehydrogenase activity				0.32	0.69	0.39		0.52	0.85		0.34	
Chitinase activity		1.63		0.32	0.69	1.57		0.52		0.32	0.68	
Hits	68	52	77	107	133	134	60	203	63	143	191	43
No hits	36	71	41	208	157	120	53	180	54	166	104	44
Total sequences	104	123	118	315	290	254	113	383	117	309	295	87
												
% Unique(hits)	34.615	57.72	34.75	66.03	54.13	47.244	46.9	47	46.15	53.722	35.3	50.57

**Table 3:** Real-time quantitative RT-PCR results of the eight randomly selected ESTs from the SSH libraries.

**Days **(post inoculation)	**Fold Change (infected/control)**							
	**Contig101**	**Contig852**	**Contig750**	**L11_67_Sh_CT**	**Contig748**	**Contig732**	**Contig710**	**Contig935**

**94-leaf-1-day**	2.2 ± 1.5	1.0	-2.7 ± 0.0	1.0	1.0	1.0	1.0	1.0
**94-leaf-3-weeks**	3.7 ± 1.7	4.2 ± 1.0	1.0	1.0	2.4 ± 2.3	1.0	1.0	-2.7 ± 1.3
**94-leaf-8-weeks**	13.6 ± 1.0	10.1 ± 1.0	17.0 ± 1.2	1.0	15.9 ± 1.4	2.1 ± 1.7	1.0	1.0
**67-leaf-1-day**	-2.4 ± 0.8	-	1.0	1.0	-2.3 ± 0.8	3.8 ± 1.4	1.0	1.0
**67-leaf-3-weeks**	1.0	2.2 ± 1.2	1.0	1.0	1.0	1.0	1.0	-12.6 ± 1.1
**67-leaf-8-weeks**	1.0	-	-2.5 ± 1.5	1.0	1.0	1.0	1.0	1.0
**94-stem-1-day**	1.0	4.2 ± 6.2	1.0	1.0	1.0	-	1.0	6.6 ± 1.0
**94-stem-3-weeks**	30.3 ± 2.7	25.6 ± 1.2	23.5 ± 2.3	2.0 ± 0.7	5.2 ± 1.7	-118.6 ± 2.9	1.0	-56.5 ± 1.2
**94-stem-8-weeks**	464.7 ± 1.0	192.7 ± 1.1	94.0 ± 1.2	1.0	262.3 ± 1.2	1.0	1.0	1.0
**67-stem-1-day**	-2.4 ± 0.5	-2.7 ± 0.3	1.0	-4.5 ± 2.2	-2.0 ± 1.8	-6.6 ± 1.2	1.0	1.0
**67-stem-3-weeks**	2.5 ± 1.6	3.7 ± 1.3	34.2 ± 1.0	1.0	11.2 ± 1.8	-3.2 ± 3.5	1.0	1.0
**67-stem-8-weeks**	-4.4 ± 1.7	1.0	11.9 ± 3.3	1.0	3.0 ± 1.6	-8.6 ± 2.2	1.0	-2.9 ± 1.34

RNA from two tissues (stem and leaf) at three stages of development (1 day, 3 weeks and 8 weeks post infection) from both resistant (67) and susceptible (94) genotypes were analyzed. Results presented here are the mean ± SD values of biological replicates. Fold differences were calculated for Ct values of infected over control RNA samples. Values for less than two-fold change were entered as (1.0). For annotation and primer sequence details, please refer to Table-4.

**Table 4:** Details of the primers used in this study.

**Sequence ID**	**Annotation**	**Sense Primer 5----3**	**Anti-sense Primer 5---3**	**Amplicon (bp)**
HLCDTotal.seq.Contig101:60-443	No hit	AAAGGGAAGTCCACACAGCC	CGAAACCACAAACTCCAAATCAAC	199
HLCDTotal.seq.Contig852:60-447	No hit	ACGCAAGAGGGAAAGAGAAG	GGGGAGCATAGTCTACTCCA	92
HLCDTotal.seq.Contig750:60-307	Osmotin-like protein TPM-1 precursor (PR P23) (Fragment)	TAATAGAGGCTTTATGGGCAGAA	CTGATGCTTACAGCTACCCTAA	100
HLCDTotal.seq.Contig748:60-265	NP24 protein precursor (Pathogenesis-related protein PR P23)	GCAGAAGATAACATCATAGTTGGT	CCCTACAGAATACTCAAGGTATTT	121
L11_67_Sh_CT_P1_F08.ab1:60-295 1 295	Mitogen-activated protein kinase kinase MAPKK	ATTGTTTACAGATGAACGGATGCG	AGGGCGTATCGGAGGAGTTC	111
HLCDTotal.seq.Contig732:60-421	Metallothionein-like protein	TGCAGCCATTTCCGCATTTG	GCTCCTCAGAGAAAGCCACC	144
HLCDTotal.seq.Contig710:60-206	SOS2-like protein kinase	AGGAAGATCTACAGAGGAGACTTT	TGGTTGGATCGAGGAGTTTCG	88
HLCDTotal.seq.Contig935:60-374	Putative metallothionein-like protein	GCTATGTTGGGACCGTTGTGA	GCCACAGGTGCAGTCAATACA	98
